# Genomic and transcriptomic insights into methanogenesis potential of novel methanogens from mangrove sediments

**DOI:** 10.1186/s40168-020-00876-z

**Published:** 2020-06-17

**Authors:** Cui-Jing Zhang, Jie Pan, Yang Liu, Chang-Hai Duan, Meng Li

**Affiliations:** 1 Shenzhen Key Laboratory of Marine Microbiome Engineering, Institute for Advanced Study, Shenzhen University, Shenzhen, China; 2 Key Laboratory of Optoelectronic Devices and Systems of Ministry of Education and Guangdong Province, College of Optoelectronic Engineering, Shenzhen University, Shenzhen, China; 3 College of Life Sciences and Oceanography, Shenzhen University, Shenzhen, China

**Keywords:** Methanogens, Metabolism, Metagenome, Metatranscriptome, Mangrove sediment

## Abstract

**Background:**

Methanogens are crucial to global methane budget and carbon cycling. Methanogens from the phylum Euryarchaeota are currently classified into one class and seven orders, including two novel methanogen taxa, *Methanofastidiosa* and *Methanomassiliicoccales*. The relative importance of the novel methanogens to methane production in the natural environment is poorly understood.

**Results:**

Here, we used a combined metagenomic and metatranscriptomic approach to investigate the metabolic activity of methanogens in mangrove sediments in Futian Nature Reserve, Shenzhen. We obtained 13 metagenome-assembled genomes (MAGs) representing one class (*Methanofastidiosa*) and five orders (*Methanomassiliicoccales*, *Methanomicrobiales*, *Methanobacteriales*, *Methanocellales*, and *Methanosarcinales*) of methanogens, including the two novel methanogens. Comprehensive annotation indicated the presence of an H_2_–dependent methylotrophic methanogenesis pathway in *Methanofastidiosa* and *Methanomassiliicoccales*. Based on the functional gene analysis, hydrogenotrophic and methylotrophic methanogenesis are the dominant pathways in mangrove sediments. MAG mapping revealed that hydrogenotrophic *Methanomicrobiale*s were the most abundant methanogens and that methylotrophic *Methanomassiliicoccales* were the most active methanogens in the analyzed sediment profile, suggesting their important roles in methane production.

**Conclusions:**

Partial or near-complete genomes of two novel methanogen taxa, *Methanofastidiosa* and *Methanomassiliicoccales*, in natural environments were recovered and analyzed here for the first time. The presented findings highlight the ecological importance of the two novel methanogens and complement knowledge of how methane is produced in mangrove ecosystem. This study implies that two novel methanogens play a vital role in carbon cycle.

Video Abstract

## Background

Methane is the second most important greenhouse gas after CO_2_. Methanogenesis is conducted by methanogens that thrive in strictly anoxic habitats. Methanogens are considered to play important roles in the global carbon cycle and climate change.

Traditionally, methanogens have been assigned to the phylum *Euryarchaeota*, and divided into two classes: class I, containing *Methanococcales*, *Methanopyrales*, and *Methanobacteriales* and class II, containing *Methanomicrobiales*, *Methanocellales*, and *Methanosarcinales* [1]. The development of high-throughput sequencing technologies has expanded the knowledge of methanogen diversity. *Methanofastidiosa* and *Methanomassiliicoccales*, two newly described novel methanogens, also fall within the phylum Euryarchaeota but do not belong to class I or class II methanogens. Beyond that, *Bathyarchaeota*, *Verstraetearchaeota*, *Hadesarchaea*, *Korarchaeota*, *Nezhaarchaeota*, *Thaumarchaeota*, and *Helarchaeota* also contain methyl-CoM reductase (MCR)-like enzymes, the key enzyme for methane metabolism, indicating that these archaea might have potentials for methane or short-chain alkane metabolisms [2–7]. Further physiological evidence is needed to confirm that notion. No pure cultures of *Methanofastidiosa* (formerly WSA2/Arc1) have been obtained but genomic data from an anaerobic wastewater treatment bioreactor have been recovered [8]. However, no genome of *Methanofastidiosa* obtained from the natural environment is available. By contrast, isolates and cultures of *Methanomassiliicoccale*s (formerly RCIII) have been obtained from the human feces, termite gut, and water treatment sludge [9–12]. Metagenome-assembled genomes (MAGs) of *Methanomassiliicoccales* have been recently recovered from diverse natural environments [4, 13], yet the details of their metabolism are limited.

Methanogens utilize three pathways, i.e., hydrogenotrophic, acetoclastic, and methylotrophic pathways, to produce methane [14, 15]. Generally, *Methanococcales*, *Methanopyrales*, *Methanobacteriales*, *Methanomicrobiales*, *Methanocellales*, and *Methanosarcinales* are hydrogenotrophs [16]. They utilize H_2_ and CO_2_ to produce methane via the methyl branch of the archaeal type Wood–Ljungdahl pathway (WLP) plus methyl-CoM reduction. *Methanosarcina* and *Methanosaeta* (*Methanosarcinales*) are the only acetoclastic methanogens known to date, which dismutate acetate to CH_4_ and CO_2_ [17]. Further, methylotrophic methanogens can be classified in two groups: *Methanosarcinales* that possess cytochromes and other methylotrophs that lack cytochromes [18]. The latter lack the *N*^5^-methyl-tetrahydromethanopterin-coenzyme M methyltransterase (MTR) and use H_2_ reduce methyl-compounds for methane production. They include *Methanosphaera* (*Methanobacteriales*), *Methanofastidiosa*, and *Methanomassiliicoccales* [19]. Analysis of *Methanofastidiosa* MAGs revealed that they possess specific methyltransferases to reduce methylated thiol for methanogenesis [8].

In different ecological environments, methanogen communities and the accompanying metabolic pathways are different. For example, hydrogenotrophic *Methanococcales* and *Methanomicrobiales*, and acetoclastic *Methanosaeta* and methylotrophic *Methanosarcinales* contribute the most to methane emission in marine sediments [20, 21]. In freshwater sediments, acetoclastic *Methanosarcinales* contribute more to methanogenesis than hydrogenotrophic *Methanobacteriales* and *Methanomicrobiales* [22]. Further, hydrogenotrophic and acetoclastic methanogens dominated the total methanogenic community in saline lake sediments with salinity less than 3.5 g L^−1^ [23]. The relative contribution of hydrogenotrophic methanogens to total methane production increases with depth in lake sediments [24]. However, little is known about the metabolic pathways and ecological roles of *Methanofastidiosa* and *Methanomassiliicoccales* in natural environments.

Mangroves are important constituents of the coastal wetlands. They could store atmospheric CO_2_ as organic matter, so-called “blue carbon,” inhabiting approximately 0.5% of the coast and contributing 10–15% to the global carbon storage [25]. However, mangrove sediment carbon does not remain stored in perpetuity. Some of organic matter are transformed to CH_4_ and returned to the atmosphere, which has the potential to partially offset blue carbon storage in mangrove sediments [26]. Previous studies using 16S rRNA gene and metagenomics demonstrated that multiple microorganisms including methanogens are widely spread across mangrove sediments [27–31]. However, the metabolic activity and relative contributions to methane production of diverse methanogens in mangroves remain unclear, especially those of *Methanofastidiosa* and *Methanomassiliicoccales*.

Mangroves are one of the major sources of CH_4_. We collected CH_4_ efflux data from literatures for 20 sites of mangrove ecosystems worldwide including Futian Mangrove Nature Reserve (FT) (Additional file 1: Table S1). FT is located in Shenzhen Special Economic Zone. While methanogenesis was not measured in this study, a previous study has reported that methane emission rates in FT range from 242 μmol m^−2^ day^−1^ or 0.242 mmol m^−2^ day^−1^ to 124 mmol m^−2^ day^−1^ [32]. In the current study, we first conducted an overall analysis based on 16S rRNA gene sequences across six mangrove ecosystems to investigate the distribution of methanogens, and the potential interactions between methanogens and other microbial lineages [33]. Then we sampled five sediment layers from FT. Previous 16S rRNA gene analysis of samples collected at the same site revealed the presence and high relative abundance of novel methanogens, including *Methanofastidiosa* and *Methanomassiliicoccales*, indicating that methanogens are a dominant archaeal group in FT mangroves [34]. We combined metagenomic and metatranscriptomic analyses to investigate the metabolic activity and relative contributions of diverse methanogens to methane production in a vertical sediment profile in mangrove ecosystem. We recovered and annotated 13 methanogen MAGs to investigate their adaption strategies to the environment. Based on the metabolic analysis and mapping results, we proposed that two novel methanogens were active for methane production and played a vital role in global carbon cycle.

## Results

### Methanogen diversity and co-occurrence network

The 16S rRNA genes sequence analysis of 78 mangrove sediment samples revealed a variety of methanogens in mangrove wetlands in southeastern China. Among the six sampling sites, methanogen abundance was the highest in Shenzhen, where they accounted for approximately 1.5% of prokaryotes (Additional file 2: Fig. S1a). The community compositions of methanogens at each site are presented in Additional file 2: Fig. S1b. *Methanofastidiosa*, *Methanosarcinales*, and *Methanomicrobiales* were widely distributed in mangrove sediments. Further, *Methanomassiliicoccales*, *Methanofastidiosa*, *Methanosarcinales*, and *Methanomicrobiales* were the four dominant methanogens in Shenzhen mangroves.

**Fig. 1:**
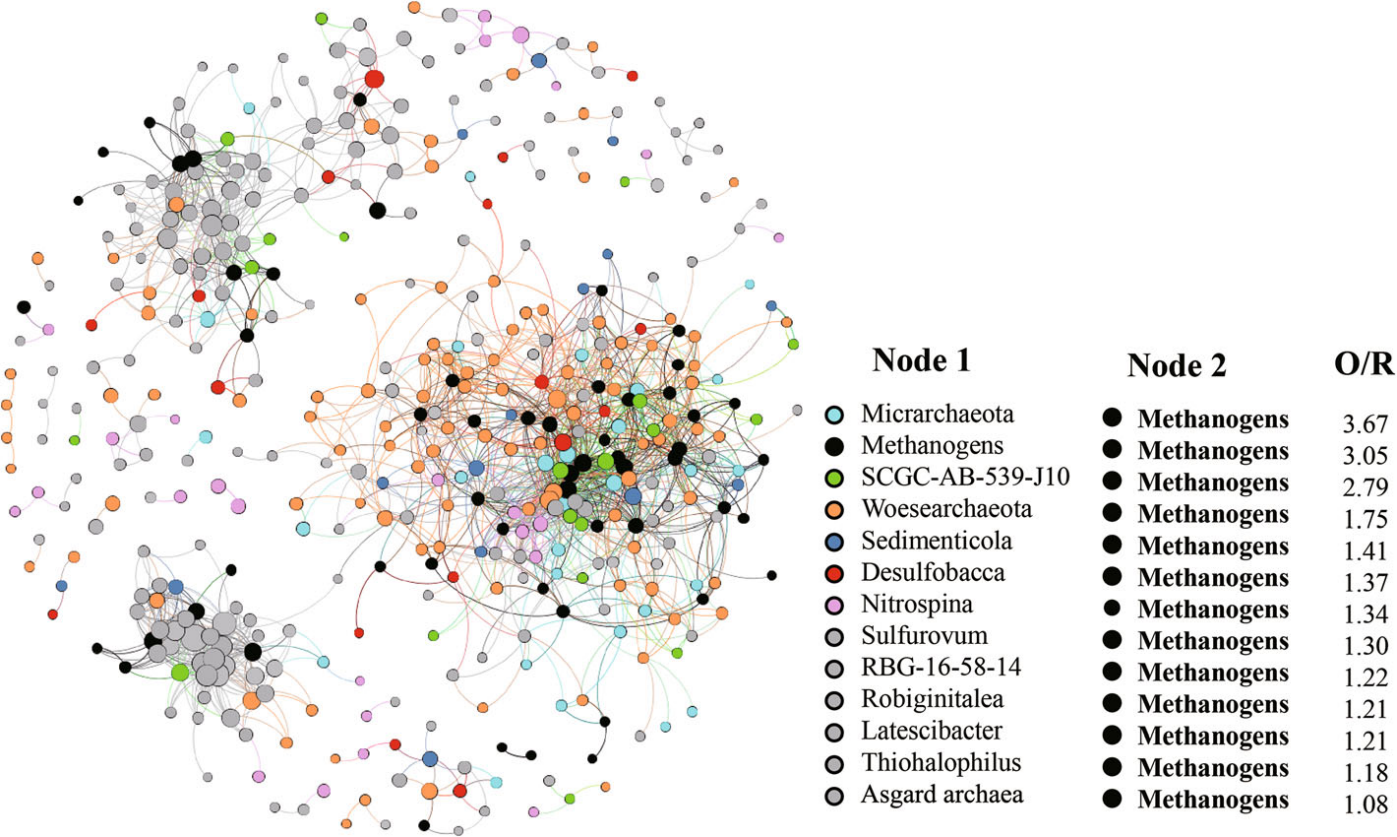
Co-occurrence network reconstructed based on 16S rRNA gene sequencing data. Different color nodes represent OTUs affiliated with different lineages. Lines (edges) connecting the nodes represent strong (*r* > 0.6) and significant (*P* < 0.01) positive correlations. O/R value is the ratio of observed to random co-occurrence incidence between two lineages (methanogens and archaeal phyla or bacterial genera). O/R values of more than 1 indicate a non-random association between two lineages

Co-occurrence network analysis based on 16S rRNA gene sequences of 78 mangrove sediment samples revealed interesting potential interactions between methanogens and other microbial taxa (Fig. 1). According to the determined O/R (observed/random incidence) ratios, *Micrarchaeota* showed the highest non-random association with methanogens. *Woesearchaeota*, *Sedimenticola*, *Desulfobacca*, and *Sulfurovum* also showed significant non-random association with methanogens.

### Genome reconstruction

De novo genomic assembly and binning of metagenome sequencing data from five layers of mangrove sediments in Futian Nature Reserve resulted in the reconstruction of 13 MAGs representing *Euryarchaeota* methanogens (Table 1). Genome size, GC content, completeness, and contamination are summarized in Table 1. The completeness of 12 MAGs ranged from 69.8 to 99.4%, while one MAG was only 50.3% complete. The contamination degree ranged from 0 to 9.6%, as assessed using CheckM (as described in “Methods” section). MAG genome sizes ranged from 0.61 to 2.33 Mbp.

**Table 1:** Characteristics of 13 methanogen MAGs reconstructed in the current study

Taxonomy	Bin ID	Size (Mbp)	Compl. (%)	Cont. (%)	Strain hetero.	Scaffolds (no.)	Genes (no.)	GC (%)	Longest scaffold (kbp)
*Methanobacteriales*	MB	0.61	50.3	0	0	149	711	49.69	13.70
*Methanocellales*	MC	0.92	86.6	2.3	60	95	1079	40.55	44.72
*Methanofastidiosa*	MF1	1.34	69.8	9.6	75	273	1713	33.53	15.53
*Methanofastidiosa*	MF2	1.17	76.0	6.2	37.5	228	1488	34.66	16.22
*Methanomassiliicoccales*	MMA1	1.98	99.2	3.4	25	107	2070	52.09	165.93
*Methanomassiliicoccales*	MMA2	1.94	98.4	2.4	50	41	1951	51.47	192.86
*Methanomicrobiales*	MM1	1.67	93.7	2.0	100	176	1941	62.18	52.51
*Methanomicrobiales*	MM2	1.41	95.3	3.3	83.33	245	1771	58.7	26.72
*Methanomicrobiales*	MM3	1.37	83.7	4.2	70	229	1686	55.47	34.61
*Methanomicrobiales*	MM4	1.64	99.4	1.0	100	80	1847	57.86	62.48
*Methanosarcinales*	MS1	1.35	78.0	2.6	75	329	1595	44.81	16.77
*Methanosarcinales*	MS2	2.33	79.4	8.2	38.46	508	2739	51.22	16.94
*Methanosarcinales*	MS3	1.89	93.6	0.7	100	258	2013	56.39	29.91

The following are shown: *Compl.* estimated completeness, *Cont.* estimated contamination, *Strain hetero.* strain heterogeneity, number of scaffolds, number of protein-coding genes, and *GC* guanine-cytosine content

### Phylogeny and relative abundances of methanogens in mangrove sediments

**Fig. 2:**
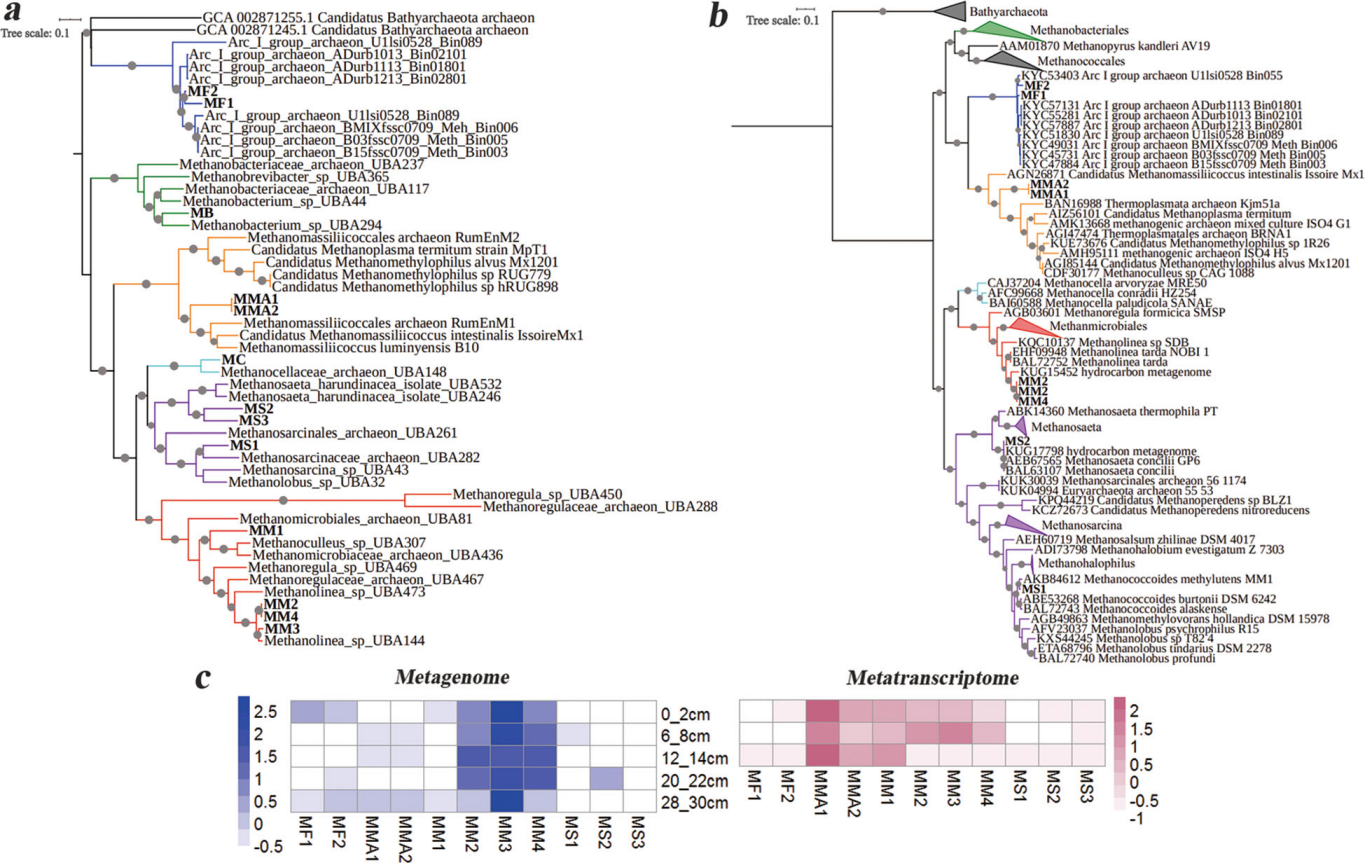
Phylogenetic trees of recovered MAGs constructed using inferred 16 ribosomal protein genes (**a**) and McrA protein sequences (**b**) using the Bathyarchaeota as the out-group. Names in bold represent MAGs in the current study. Bootstrap values were calculated via non-parametric bootstrapping with 100 replicates, and are represented by gray circles in different sizes. The scale bar indicates 10% estimated phylogenetic divergence. **c** The relative abundances (metagenome, RPKM, blue) and expression levels (metatranscriptome, RPKM, red) of MAGs affiliated with four dominant methanogen lineages (MF, MMA, MM, and MS)

To identify MAG lineages, we constructed phylogenetic trees based on a concatenated set of 16 ribosomal proteins (Fig. 2a), McrA (methyl-coenzyme M reductase alpha subunit) protein sequences (Fig. 2b), and 16S rRNA genes (Additional file 3: Fig. S2). Four MAGs represented two newly described lineages, MF1 and MF2, clustered within the class *Methanofastidiosa*, a class distinct from other Euryarchaeota and MMA1 and MMA2, clustered with *Methanomassiliicoccales*. The remaining 9 MAGs corresponded to the traditional methanogen lineages within class I or class II methanogens: MB, belonging to the order *Methanobacteriales*; MC from the order *Methanocellales*; MM1, MM2, MM3, and MM4 from the order *Methanomicrobiales*; and MS1, MS2, and MS3 from the order *Methanosarcinales*.

Nine *mcrA* gene sequences were retrieved from 13 MAGs (Fig. 2b). A phylogenetic tree constructed using the corresponding McrA sequences was consistent with the tree constructed using conserved ribosomal proteins. The *mcrA* gene sequences were not retrieved from the MB and MC genome bins, probably because of the incompleteness of the genomes. McrA from MM was affiliated with the sequences from the genus *Methanolinea*; McrA from MS1 clustered with the sequences from the genus *Methanococcoides*; and McrA from MS2 clustered with the sequences from the genus *Methanosaeta*.

To reveal the relative importance of different methanogens in a vertical mangrove sediment profile, the relative abundance and activity of methanogens were next evaluated (Fig. 2c). Members of MM were the most abundant group of methanogens in all layers. Although MMA MAGs were not abundant compared to MM MAGs, they were the most active group in all layers according to the metatranscriptomic analysis. MF and MS coexisted with other methanogens in all layers, but their relative abundance and activity were relatively low.

### Relative importance of the three major metabolic pathways for methanogenesis in mangrove sediments

Quantitative PCR analysis revealed a dramatically different abundance of methanogens in the five sampled sediment layers. Indeed, the copy numbers of the *mcrA* gene ranged from 10^5^ to 10^6^ gene copies per gram sediment, with the highest abundance in the 6–8 cm layer (Additional file 4: Fig. S3).

**Fig. 3:**
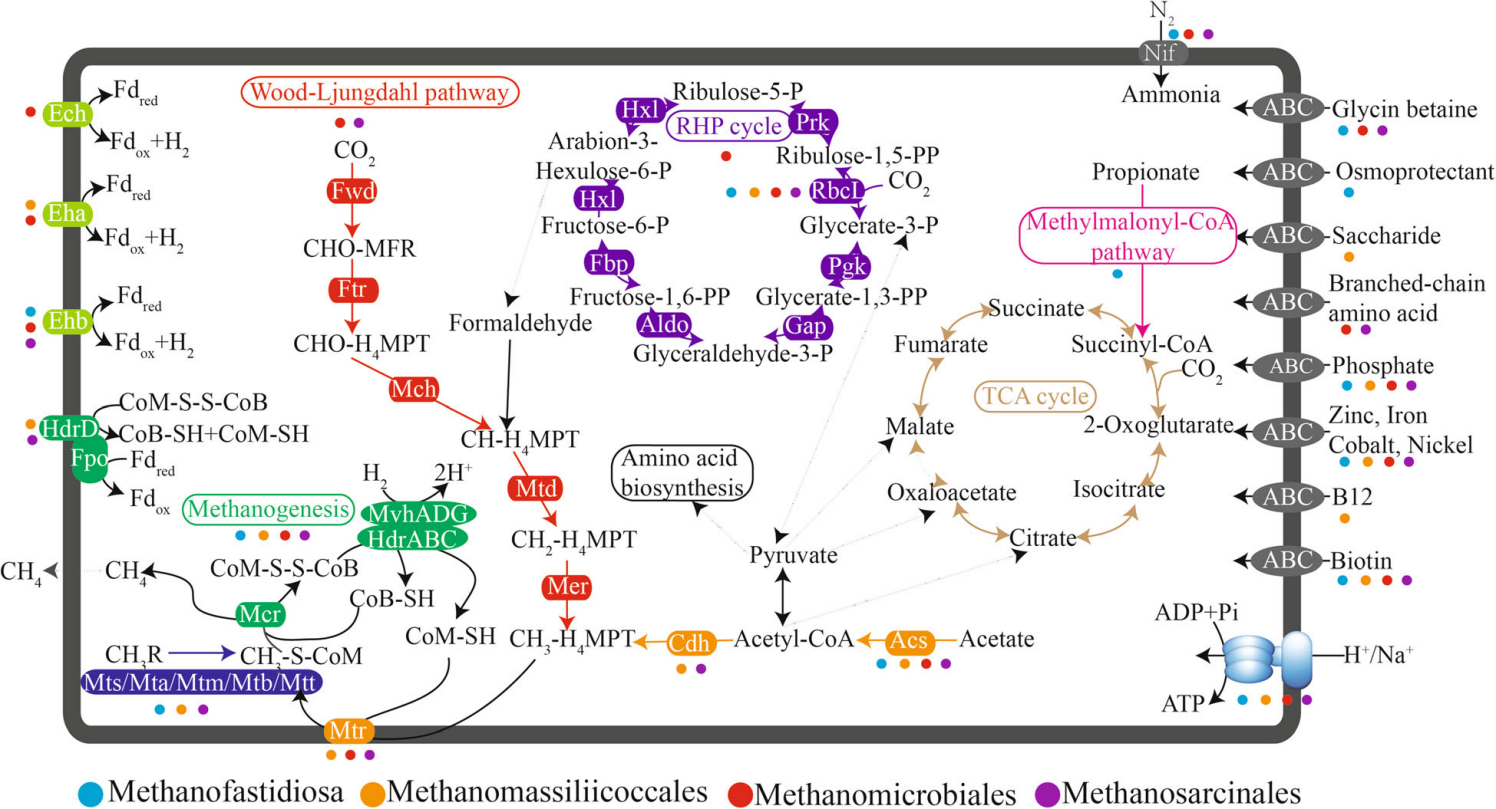
Predicted metabolic pathways in the four dominant methanogen lineages (MF, MMA, MM, and MS), based on analysis of 11 MAGs. Genes involved in the metabolism of carbon, nitrogen, and hydrogen; energy conservation; and various transporters are shown in different colors. Predicted proteins in the figures are listed in Additional file 6: Table S2

MAG annotation revealed a diverse metabolic potential for methanogenesis. Three complete metabolic pathways (hydrogenotrophic, acetoclastic, and methylotrophic methanogenesis) were identified (Fig. 3). MM and MS MAGs shared the hydrogenotrophic methanogenesis pathway. They possessed genes encoding conserved core enzymes of hydrogenotrophic methanogenesis, including Fwd, Ftr, Mch, Mtd, Mer, Mtr, and Mcr. MS MAGs showed potential for acetoclastic methanogenesis. They contained genes encoding Acs, Cdh, Mtr, and Mcr, enzymes for the utilization of acetate. Further, MF, MMA, and MS MAGs contained genes encoding methyl-compound methyltransferase, i.e., Mts, Mta, Mtm, Mtb, and Mtt. This indicated a potential for methane production via the methylotrophic pathway. Because of the genome incompleteness, no complete methanogenesis pathways were identified in MB and MC MAGs.

**Fig. 4:**
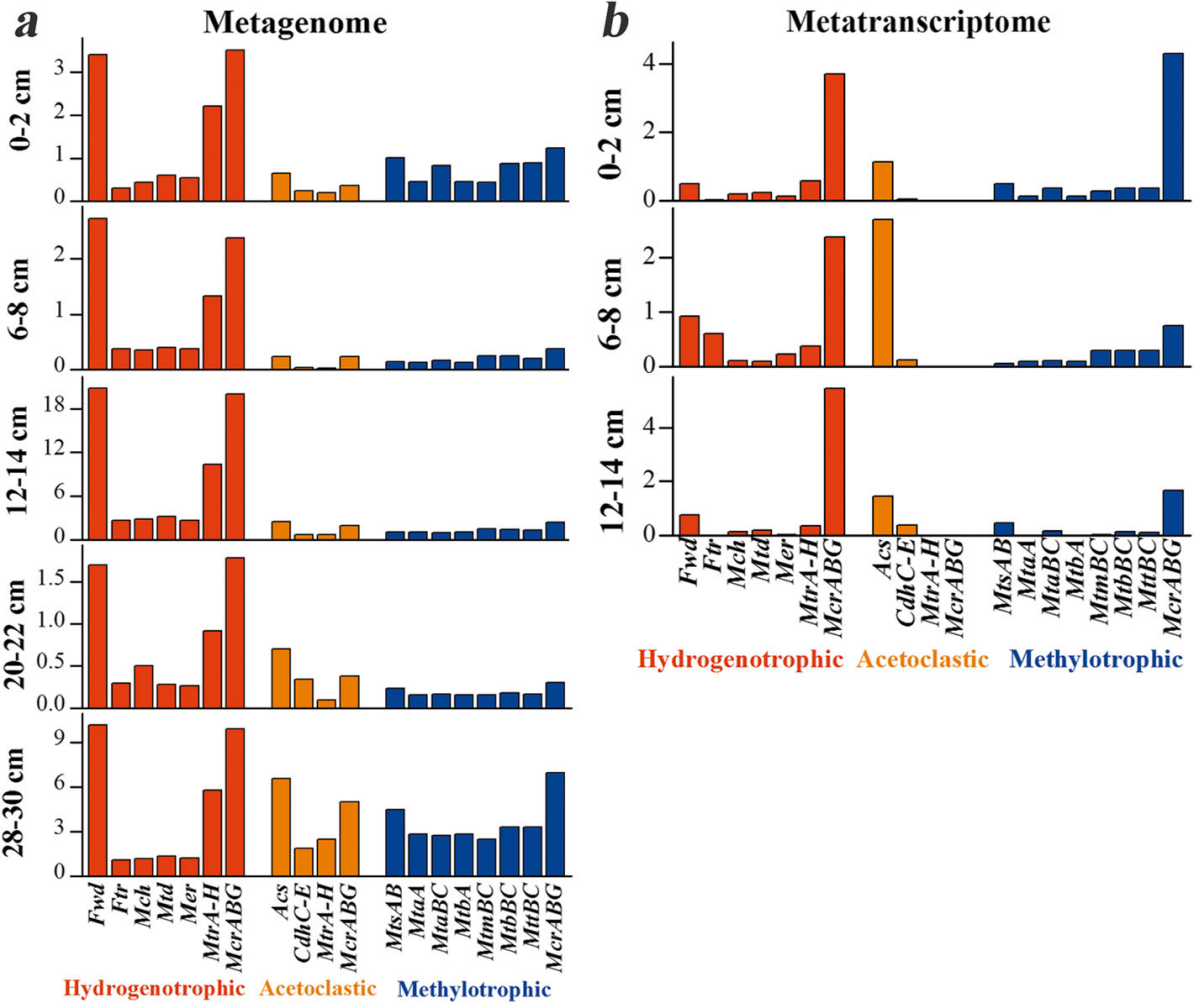
Relative abundances (**a**) and expression levels (**b**) of genes involved in three methanogenesis pathways. The relative abundances and expression levels were evaluated based on FPKM values. The three metabolic pathways are represented by different colors (red, hydrogenotrophic; orange, acetoclastic; and blue, methylotrophic)

To evaluate the relative importance of the three metabolic pathways for methane generation in the vertical mangrove sediment profile, the relative abundances and expression of the relevant genes were evaluated (Fig. 4). The functional analysis revealed that the relative abundances of genes associated with autotrophic hydrogenotrophic methanogenesis were higher than those of heterotrophic acetoclastic or methylotrophic methanogenesis pathways in all layers. Further, genes involved in hydrogenotrophic and methylotrophic methanogenesis were highly expressed.

Next, the relative abundances and expression of genes from different MAGs were evaluated (Additional file 5: Fig. S4). Genes encoding enzymes from the hydrogenotrophic pathway in MM were highly abundant and expressed in all layers. Although the H_2_–dependent methylotrophic pathway in MF was expressed, the metabolic activity was relatively low. The MMA genes *mts*, *mta*, *mtb*, *mtt*, and *mcr*, encoding enzymes involved in the reduction of methyl compounds, were highly expressed.

## Discussion

Methane emission in mangroves is strongly affected by anthropogenic activities including aquaculture and sewage [35]. Most pristine mangroves showed low CH_4_ efflux rate, while mangroves with human disturbances showed significantly higher CH_4_ efflux rate including FT mangroves (Additional file 1: Table S1). In the current study, we recovered and annotated 13 methanogen MAGs including two novel methanogen taxa, *Methanofastidiosa* and *Methanomassiliicoccales* from FT mangrove sediments. We aimed to show the relative importance of the novel methanogens to methane production. Our results showed that, according to transcript levels, *Methanomassiliicoccale*s were the most active methanogens. These observations implied that two novel methanogens make contributions for methane production and play a vital role in carbon cycle.

In the current study, we identified and analyzed diverse methanogens in mangrove sediments. Copies of the *mcrA* gene ranged from 10^5^ to 10^6^ per gram sediment. The low gene copies might be due to primer pair we used for quantitative PCR. Since there were no generally recognized primers for novel methanogens, we used the primers mlas-mod-F and mcrA-rev-R [36]. This primer pair targets traditional methanogens and has low specificity to the novel methanogens including *Methanofastidiosa* and *Methanomassiliicoccales*, potentially underestimate the real abundance of methanogens. Therefore, the *mcrA* gene abundance could be higher if *Methanofastidiosa* and *Methanomassiliicoccales* were taken into account. The highest copy number was observed in the 6–8-cm layer (Additional file 4: Fig. S3). Previous studies similarly reported the highest methane production at the sediment surface [37]. The vertical variations of methanogen abundance are thought to be associated with the changing physiochemical factors in the sediment [24, 38]. The total organic carbon (TOC) concentration in the 0–2-cm layer was approximately 1.78 mg/g and decreased to approximately 0.96 mg/g in the 28–30-cm layer (Additional file 4: Fig. S3). The total nitrogen (TN) concentration also decreased, from 1.61 mg/g at the surface to 0.63 mg/g in deeper layers (Additional file 4: Fig. S3). Since large amounts of terrestrial and riverine nutrients reach and accumulate in the estuary environment, the top layers of the intertidal sediment are characterized by high organic matter content, which provides a suitable environment for the growth of methanogens [25].

We observed that the expression of genes involved in hydrogenotrophic and methylotrophic methanogenesis in the top three layers was high (Fig. 4), indicating that these two pathways are metabolically active and greatly contribute to the methane production in mangrove sediments. This was in agreement with a report that hydrogenotrophic and methylotrophic methanogenesis dominate in coastal sediments [37]. Methylotrophic methanogens might coexist with sulfate-reducing bacteria (SRB) in sulfate-rich environments because of their exclusive utilization of and stronger affinity for methyl compounds. Co-occurrence of methanogens and *Sedimenticola*, *Desulfobacca*, and *Sulfurovum*, which belong to SRB, detected in the current study supports this notion [39–42]. Methyl compounds such as trimethylamine (TMA) contribute 35–90% of the methane production in coastal sediments [43], which could explain why methylotrophic methanogens play an important role in methane production in mangroves. We also showed that hydrogenotrophic methanogenesis is more abundant and active than methylotrophic methanogenesis. Since mangrove sediments are rich in organic carbon, hydrogenotrophic methanogens could consume H_2_ and cooperate with syntrophic microbes to degrade short-chain fatty acids [44, 45]. Co-occurrence analysis also revealed a significant non-random association of methanogens and *Woesearchaeota*. The H_2_ production and consumption by *Woesearchaeota* and methanogens might explain this co-occurrence pattern, indicating a high possibility of a syntrophic relationship [46, 47].

We observed a low relative abundance and expression of genes involved in the acetoclastic pathway. This might be explained by that acetate is used by SRB. Salinity plays an important role in regulating methanogenic community [48]. Sulfate concentration is 1.29–2.77 g per kilogram dry weight in Futian mangroves [49]. High levels of salinity/sulfate favor SRB. Due to higher affinity for acetate, SRB have a thermodynamic advantage over acetotrophic methanogens. Acetoclastic methanogens usually dominate methane production in freshwater environments, such as anaerobic digesters, rice fields, and freshwater wetlands [50, 51]. Although 20–22 cm and 28–30 cm were sampled in the current study, we were unable to obtain transcriptomic data for these two layers because of the low quality of RNA extracted for sequencing (data not shown). This might be associated with the slow growth rates of microorganisms in undisturbed deep environments [52].

MF, MMA, MM, and MS were the four dominant methanogen lineages identified in the current study. This was consistent with previous 16S rRNA gene and *mcrA* sequencing-based identification of *Methanomicrobiales* and *Methanosarcinales* in coastal sediments [53–55]. Mangrove sediments in the Futian Natural Reserve are characterized by high sulfate concentrations [49], suggesting that adaptation to high salinity is important for mangrove dwelling. Therefore, methanogens should maintain an osmotic pressure equivalent to that of their surroundings, which might be achieved by the accumulation of organic osmotic solutes [56]. Indeed, genes for an organic solute transporter induced by glycine-betaine were identified in MF, MM, and MS MAGs. MF MAGs also encode an osmoprotectant transporter.

Notably, MM was the most abundant group of methanogens in all layers. Further, MM genes encoding enzymes from the hydrogenotrophic pathway were highly abundant and expressed in all layers (Additional file 5: Fig. S4), suggesting MM is the predominant group of hydrogenotrophic methanogens in mangrove sediments. This might be explained by a large number of electron transporters encoded in their genomes, which is advantageous for microbial adaption to low substrate (H_2_) environments [57]. In addition, MM encoded multiple membrane-bound hydrogenases, including Ech, Eha, and Ehb (Fig. 3 and Additional file 6: Table S2). Finally, MM possessed a complete gene set for the reductive hexulose-phosphate (RHP) pathway for autotrophic carbon fixation. This pathway has been proposed recently and is expected to be widely distributed among *Methanomicrobiales* [58].

In the current study, the partial MF genomes from a natural environment were reported for the first time. Members of MF are distinguished from the traditional class I and class II methanogens by the lack of genes encoding enzymes for conventional CO_2_ reduction. Members of MF lack the MTR complex and use H_2_ as a reductant for methanogenesis. Specifically, MF MAGs harbor genes encoding substrate-specific methyltransferases for multiple methyl compounds, including Mts, Mta, Mtb, and Mtt (Fig. 3 and Additional file 6: Table S2). They also harbor genes for heterodisulfide reductase (*hdrABC*)/[Ni-Fe] hydrogenase (*mvhADG*) complex for heterodisulfide coenzyme B coenzyme M (CoB-S-S-CoM) reduction and H_2_ oxidation. Further, they contain genes encoding the membrane-bound energy-conserving hydrogenase (Ehb) to generate H_2_. These two processes could be connected to H_2_ cycling. The *mcr* genes from MF were expressed. These observations suggested that MF could produce methane via H_2_ reduction of multiple methyl compounds in mangrove sediments, as opposed to solely relying on methylated thiols, as described previously [8]. That might be because mangrove plants produce methanol and methylamines that could be utilized by methylotrophic methanogens.

Although the H_2_-dependent methylotrophic pathway in *Methanofastidiosa* was expressed at 6–8 cm, the metabolic activity was relatively low in mangrove sediments. This might be due to that *Methanofastidios*a are heterotrophic methanogens. They generally dwell in eutrophic environments, such as wastewater treatment sludge and digesters [59, 60]. These microorganisms require exogenous organic carbon (acetate or propionate) as a carbon source for growth as they lack genes encoding the carbon fixation pathway (Fig. 3). Organic matter contents in the sampled environments were not high enough for growth of *Methanofastidiosa*.

*Methanomassiliicoccales* have been isolated and enriched from human feces, termite gut, and an anaerobic digester [10–12, 61]. Recently, *Methanomassiliicoccales* MAGs have been recovered from the natural environment [4, 13]. In the current study, near-complete genomes of *Methanomassiliicoccales* from the mangrove sediments were analyzed for the first time. Similar to MF, MMA MAGs harbor a pathway for H_2_ reduction of methyl compounds (methylsulfides, methanol, and methylamines) (Fig. 3 and Additional file 6: Table S2), as reported in the previously described *Methanomassiliicoccales* genomes and validated by physiological experiments [18]. The MAGs also contained genes for the HdrABC/MvhADG complex for H_2_ oxidation. There is evidence for an additional coupling of ferredoxin and heterodisulfide in *Methanomassiliicoccales*, operated by the association of F_420_H_2_ hydrogenase (Fpo) and a second heterodisulfide reductase (HdrD) [62]. Transcripts of the MMA *mts*, *mta*, *mtb*, *mtt*, and *mcr* genes encoding proteins for the reduction of methyl compounds were detected, suggesting that MMA might contribute to methane production via the H_2_-dependent methylotrophic methanogenesis pathway. This could be explained by the availability of a variety of methylotrophic substrates (methylsulfides, methanol, and methylamines) in mangrove sediments.

According to metatranscriptomic analysis in the current study, MMA were the most active group in all layers, suggesting that MMA are well adapted to the fluctuating environment of mangrove sediments. Indeed, prevalence of *Methanomassiliicoccale*s in coastal sediments has been reported [31, 54], indicating their importance for methane production in natural environments. MMA MAGs contained genes encoding oligo- and monosaccharide transporters, which might illustrate adaptation to a heterotrophic lifestyle (Fig. 3). Nevertheless, isolates from natural environments should be obtained and analyzed to validate their inferred physiological characteristics [63].

## Conclusions

In conclusion, *Methanofastidiosa*, *Methanomassiliicoccales*, *Methanomicrobiales*, and *Methanosarcinales* were identified as the four dominant and potential methanogens in mangrove sediments. To the best of our knowledge, metabolic pathways utilized by the two novel methanogens *Methanofastidiosa* and *Methanomassiliicoccales* in the natural environment were here analyzed for the first time. Analysis of genes involved in methanogenesis suggested that the hydrogenotrophic and methylotrophic pathways contributed the most to the methane production in mangrove ecosystems. Based on the metagenomic and metatranscriptomic data, *Methanomicrobiales* and *Methanomassiliicoccales* are the most abundant and active methanogens, respectively. Collectively, the current study provides insights into the relative importance of diverse methanogens for methane production and advances the understanding of different methanogenesis pathways in mangrove ecosystems. This study implies that two novel methanogens play important roles in global carbon cycle.

## Methods

### Diversity and community network analysis of methanogens in mangrove sediments

Prokaryotic 16S rRNA genes in 78 sediment samples from 6 mangrove ecosystems across southern China were previously sequenced using the primer pair 515F/806R, and raw reads were processed as previously described [33]. Operational taxonomic units (OTUs) were picked at 97% cutoff using QIIME scripts [64]. Representative sequences of each OTU were assigned according to the SILVA SSUPara database (v132) [65]. Sequences belonging to methanogens were extracted to make “Methanogens_OTU table.” Diversity of methanogens in mangrove sediments was calculated based on the “Methanogen_OTU table.” Abundant and ubiquitous OTUs whose abundance > 0.001% of total sequences and occurrence in more than one sample were selected to make “Core_OTU table.” To explore the co-occurrence patterns between methanogens and other microbes, network analysis was conducted by calculating the correlations based on the “Core_OTU table” [66]. The obtained network reflected positive correlations (edges) among OTUs (nodes) with Spearman’s *ρ* > 0.6 and FDR-adjusted *p* value < 0.01. The network contained 3548 nodes and 36,248 edges. The random and observed incidence of co-occurrence patterns between methanogens and archaeal phyla or bacterial genera were calculated [67]. Only OTUs of methanogens and taxa with the O/R (observed/random incidence) ratio above 1 were retained for visualization. The network was constructed, characterized, and visualized using R packages (vegan and igraph) and the software gephi [68, 69].

### Sample collection, nucleic acid extraction, and metagenome/metatranscriptome sequencing

A 32-cm sediment core, vertically stratified at 2-cm–depth intervals (32 cm in total), was collected for DNA extraction at an intertidal mudflat in the Futian Nature Reserve of Shenzhen (22.53°N, 114°E) in April 2017. A description of physicochemical parameters’ measurement can be found in previous study [34]. Samples from the 0–2, 6–8, 12–14, 20–22, and 28–30-cm layers were selected for metagenomic analysis. For the above fiver layers, genomic DNA was extracted from 5 g of wet sediment using DNeasy PowerSoil kit (Qiagen, Germany), according to the manufacturer’s instructions, and stored at – 20 °C. Metagenomic sequence data were generated using Illumina Hiseq 2000 instrument at Novogene Bioinformatics Technology Co., Ltd. (Tianjin, China). Approximately 110 Gbp (2 × 150 bp paired-end reads) of raw sequence data were generated for each sample.

The samples for metatranscriptomic analysis were collected at the same site as that used for metagenomic analysis from the 0–2, 6–8, and 12–14-cm layers in April 2018. The sediments were preserved immediately after collection in the LifeGuard Soil Preservation Solution (Qiagen) to prevent RNA degradation. Total RNA was isolated from wet sediment (4–20 g) using an RNeasy PowerSoil Total RNA kit (Qiagen), according to the manufacturer’s protocol. Genomic DNA was removed by using TURBO DNA-free kit (Ambion, USA), and the remaining RNA was concentrated and purified by using the RNeasy MinElute Kit (Qiagen). The extracted RNA (approximately 3 μg per sample) was paired-end sequenced using Illumina Hiseq 2000 instrument at Novogene (Tianjin, China). Approximately 8 Gbp (2 × 150 bp paired-end reads) of raw sequence data were generated for each sample.

### Determination of methanogen abundances in the sediment layers

Abundances of the *mcrA* gene in the five sediment layers were determined by quantitative PCR using the primers mlas-mod-F and mcrA-rev-R [36], and an iCycler iQ 5 thermocycler (Bio-Rad, USA). The reaction volume was 25 μl. Each reaction contained 12.5 μl of 2 × SYBR® Premix Ex TaqTM (Takara Biotechnology, Japan), 0.5 μl of each primer (10 μM), and 2 μl of diluted DNA template (1–10 ng). The amplification program consisted of 30 cycles of 30 s at 95 °C, 45 s at 55 °C, and 30 s at 72 °C. Standard curves were generated using 10-fold serial dilutions of a plasmid containing the *mcrA* gene fragments. The PCR efficiency ranged between 90 and 100%, with *R*^2^ value of 0.99.

### De novo assembly, binning, and annotation

Metagenomic datasets generated for the five layers of mudflat sediments were used in a combined assembly to recover genomes. The raw reads were dereplicated and trimmed using Sickle (https://github.com/najoshi/sickle). High-quality metagenomic sequences were de novo assembled using IDBA-UD [70] with the following parameters: -mink 65, -maxk 145, and -step 10. Genome binning of the assembled fragments was done using MetaBAT [71]. Partial and near-complete genomes were recovered after binning. Manual refining of MAGs was performed using Anvi’o to remove contaminating contigs [72]. The completeness, contamination, and strain heterogeneity of MAGs were evaluated by using CheckM (version 1.0.5) [73]. Thirteen MAGs representing one class (*Methanofastidiosa*) and five orders (*Methanomassiliicoccales*, *Methanobacteriales*, *Methanocellales*, *Methanomicrobiales*, and *Methanosarcinales*) of methanogens were selected for further analysis. The MAGs were translated by Prodigal using the “-p meta” parameters [74]. For each predicted coding sequence (CDS), protein function was annotated using the KEGG server (BlastKOALA) and eggNOG-mapper [75, 76].

### Phylogenetic analysis

The concatenated ribosomal protein tree was generated as described elsewhere [77]. Briefly, several reference genomes of methanogens from the phylum *Euryarchaeota* were downloaded from the NCBI (https://www.ncbi.nlm.nih.gov/) and IMG-M (https://img.jgi.doe.gov/cgi-bin/m/main.cgi) databases. The information for reference genomes is provided in Additional file 7: Table S3. For the analysis, genes for 16 ribosomal proteins (ribosomal proteins L2–L6, L14–L16, L18, L22, L24, S3, S8, S10, S17, and S19) were used [46]. The 16 ribosomal proteins were aligned independently using MUSCLE [78]. The amino acid alignments were then used for phylogenetic tree construction using FastTree with default parameters [79].

Genes annotated as 16S rRNA and *mcrA* were extracted against the SILVA SSU132 [65] and *mcrA* database downloaded from FunGene (http://fungene.cme.msu.edu/index.spr), respectively. Reference sequences of the 16S rRNA and *mcrA* genes of methanogens from the phylum Euryarchaeota were downloaded from NCBI (https://www.ncbi.nlm.nih.gov/) and FunGene, respectively. The 16S rRNA gene sequences were aligned using SINA [80]. The amino acid sequences of predicted McrA proteins were aligned using ClustalW [81]. Phylogenetic trees for the 16S rRNA gene and McrA protein sequence were constructed in FastTree using default parameters [79]. The trees were visualized by using iTOL [82], and rooted using the Bathyarchaeota as an out-group.

### Gene abundance and expression

Raw reads generated after metatranscriptomic sequencing were filtered using SortMeRna to remove tRNA and rRNA sequences [83]. Reads per kilobase of transcript per million mapped reads (RPKM) for genomic and transcriptomic reads were calculated to determine the relative abundance and expression activity of MAGs, respectively. Relative abundances were determined by mapping to methanogen MAGs using Bowtie [84]. Expression activity was determined by mapping non-rRNA transcripts to methanogen MAGs using BWA-MEM [85]. The abundance and expression of key genes encoding enzymes involved in methanogenesis were calculated by mapping raw metagenome and metatranscriptome reads to each CDS of MAGs using fragments per kilobase of transcript per million mapped reads (FPKM), respectively.

## Supplementary information

**Additional file 1: Table S1.** Range of CH_4_ emission from different mangrove ecosystems across the world.

**Additional file 2: Figure S1.** Relative abundance of methanogens in prokaryotes (a) and community composition of methanogens (b) among 6 mangroves across southeastern China.

**Additional file 3: Figure S2.** Phylogenetic trees of recovered MAGs using 16S rRNA gene sequences. Each name in bold represent a MAG. Bootstrap values were calculated via non-parametric bootstrapping with 100 replicates, and are represented by grey circles in different sizes. The scale bar indicates 10% estimated phylogenetic divergence.

**Additional file 4: Figure S3.** Physiochemical properties and *mcrA* gene copies in five layers of mangrove sediments.

**Additional file 5: Figure S4.** The relative abundances (metagenome, FPKM, blue) and expression levels (metatranscriptome, FPKM, red) of genes involved for methanogenesis with affiliation to four dominant methanogens (MF, MMA, MM and MS) at five layers in mangrove sediments.

**Additional file 6: Table S2.** Genes of the recovered MAGs related to methanogenesis, energy conservation, carbon, nitrogen, sulfate, amino acid and ABC transporter.

**Additional file 7: Table S3.** Reference genomes used to build the methanogens ribosomal protein tree (Fig. 1a).

## Data Availability

The genome bins generated and analyzed during the current study are available in the NCBI and can be viewed under Project PRJNA587831. The metagenome and transcriptome data can be viewed in NODE (http://www.biosino.org/node) by pasting the accession (OEP000712) into the text search box or through the URL: http://www.biosino.org/node/project/detail/OEP000712.

## Abbreviations

MAGs: Metagenome-assembled genomes
MCR: Methyl-CoM reductase
WLP: Wood–Ljungdahl pathway
MTR: *N*^5^-methyl-tetrahydromethanopterin-coenzyme M methyltransterase
*mcrA*: Methyl-coenzyme M reductase alpha subunit
FT: Futian Mangrove Nature Reserve
O/R: Observed/random incidence
TOC: Total organic carbon
TN: Total nitrogen
RHP: Reductive hexulose-phosphate
Hdr: Heterodisulfide reductase
Mvh: [Ni-Fe] hydrogenase
CoB-S-S-CoM: Heterodisulfide coenzyme B coenzyme M
Ehb: Energy-conserving hydrogenase
Fpo: F_420_H_2_ hydrogenase
Acs: Acetyl-CoA synthetase
Cdh: Carbon monoxide dehydrogenase
CDS: Coding sequence
RPKM: Reads per kilobase of transcript per million mapped reads
FPKM: Fragments per kilobase of transcript per million mapped reads
