# Supplementary material for: Genomic and transcriptomic insights into methanogenesis potential of novel methanogens from mangrove sediments
Source: Microbiome. 2020 Jun 17;8:94. doi: 10.1186/s40168-020-00876-z (PMC7302380; doi:10.1186/s40168-020-00876-z)
Supplement: Supplementary file 2 — Additional file 1: Table S1. Range of CH4 emission from different mangrove ecosystems across the world. [file 40168_2020_876_MOESM1_ESM.docx]

**Table S1**

| Site of mangrove ecosystems | Average CH_4_ flux rate  (μmol m^−2^ day^−1^) | Reference |
| --- | --- | --- |
| Queensland, Australia | 40.3 - 1047.1 | ([Rosentreter et al 2018](#_ENREF_19)) |
| Evans Head, Australia | 270 | ([Santos et al 2019](#_ENREF_20)) |
| Westernport Bay, Victoria, Australia | 750 | ([Livesley and Andrusiak 2012](#_ENREF_16)) |
| Jaguaribe River, Ceará, Brazil | 300 - 1200 | ([Queiroz et al 2019](#_ENREF_18)) |
| Sepetiba Bay, Rio de Janeiro, Brazil | 2.7 - 3.1 | ([Marinho et al 2012](#_ENREF_17)) |
| Yucatán Peninsula, Mexico | 2.3 - 31000 | ([Chuang et al 2016](#_ENREF_7), [Chuang et al 2017](#_ENREF_8)) |
| Ciénaga Grande de Santa Marta, Colombia | 0 - 47355 | ([Konnerup et al 2014](#_ENREF_13)) |
| Naples Bay, Florida, USA | 20 - 140 | ([Cabezas et al 2018](#_ENREF_1)) |
| Ouemo, New Caledonia, France | 39.4 - 428.1 | ([Jacotot et al 2019](#_ENREF_12)) |
| Eastern coast of the Red Sea | 0.9 - 13.3 | ([Sea et al 2018](#_ENREF_21)) |
| Tanzanian | 10 - 350 | ([Kristensen et al 2008](#_ENREF_14)) |
| Honda Bay, Palawan, Philippines | 109.2 - 183.4 | ([Castillo et al 2017](#_ENREF_2)) |
| Sundarban, Bay of Bengal | 31.6 - 6230 | ([Das et al 2018](#_ENREF_9), [Dutta et al 2017](#_ENREF_10)) |
| Eastern coast of India | 120 - 3450 | ([Chauhan et al 2015](#_ENREF_3)) |
| Muthupet, South India | 1186 - 2346 | ([Krithika et al 2008](#_ENREF_15)) |
| North Sulawesi, Indonesia | −8.4 - 14.6 | ([Chen et al 2014](#_ENREF_6)) |
| Dongzhaigang, Hainan, China | 2040 - 7230 | ([He et al 2019](#_ENREF_11)) |
| Zhangjiangkou, Fujian, China | 17.5 - 2966.2 | ([Zheng et al 2018](#_ENREF_22)) |
| **Futian, Shenzhen, China** | **242.4 - 124046** | **(**[**Chen et al 2010**](#_ENREF_4)**)** |
| Sai Keng, Hong Kong, China | 285.8 - 5770.3 | ([Chen et al 2011](#_ENREF_5)) |

**References**

Cabezas A, Mitsch WJ, MacDonnell C, Zhang L, Bydałek F, Lasso A (2018). Methane emissions from mangrove soils in hydrologically disturbed and reference mangrove tidal creeks in southwest Florida. *Ecological Engineering* **114:** 57-65.

Castillo JAA, Apan AA, Maraseni TN, Salmo SG (2017). Soil greenhouse gas fluxes in tropical mangrove forests and in land uses on deforested mangrove lands. *Catena* **159:** 60-69.

Chauhan R, Datta A, Ramanathan AL, Adhya TK (2015). Factors influencing spatio-temporal variation of methane and nitrous oxide emission from a tropical mangrove of eastern coast of India. *Atmos Environ* **107:** 95-106.

Chen GC, Tam NF, Ye Y (2010). Summer fluxes of atmospheric greenhouse gases N2O, CH4 and CO2 from mangrove soil in South China. *Sci Total Environ* **408:** 2761-2767.

Chen GC, Tam NFY, Wong YS, Ye Y (2011). Effect of wastewater discharge on greenhouse gas fluxes from mangrove soils. *Atmos Environ* **45:** 1110-1115.

Chen GC, Ulumuddin YI, Pramudji S, Chen SY, Chen B, Ye Y *et al* (2014). Rich soil carbon and nitrogen but low atmospheric greenhouse gas fluxes from North Sulawesi mangrove swamps in Indonesia. *The Science of the total environment* **487:** 91-96.

Chuang P-C, Young MB, Dale AW, Miller LG, Herrera-Silveira JA, Paytan A (2016). Methane and sulfate dynamics in sediments from mangrove-dominated tropical coastal lagoons, Yucatán, Mexico. *Biogeosciences* **13:** 2981-3001.

Chuang PC, Young MB, Dale AW, Miller LG, Herrera-Silveira JA, Paytan A (2017). Methane fluxes from tropical coastal lagoons surrounded by mangroves, Yucatán, Mexico. *Journal of Geophysical Research: Biogeosciences* **122:** 1156-1174.

Das S, Ganguly D, Chakraborty S, Mukherjee A, Kumar De T (2018). Methane flux dynamics in relation to methanogenic and methanotrophic populations in the soil of Indian Sundarban mangroves. *Marine Ecology* **39:** e12493.

Dutta MK, Bianchi TS, Mukhopadhyay SK (2017). Mangrove Methane Biogeochemistry in the Indian Sundarbans: A Proposed Budget. *Frontiers in Marine Science* **4**.

He Y, Guan W, Xue D, Liu L, Peng C, Liao B *et al* (2019). Comparison of methane emissions among invasive and native mangrove species in Dongzhaigang, Hainan Island. *The Science of the total environment* **697:** 133945.

Jacotot A, Marchand C, Allenbach M (2019). Biofilm and temperature controls on greenhouse gas (CO2 and CH4) emissions from a Rhizophora mangrove soil (New Caledonia). *The Science of the total environment* **650:** 1019-1028.

Konnerup D, Betancourt-Portela JM, Villamil C, Parra JP (2014). Nitrous oxide and methane emissions from the restored mangrove ecosystem of the Ciénaga Grande de Santa Marta, Colombia. *Estuarine, Coastal and Shelf Science* **140:** 43-51.

Kristensen E, Flindt MR, Ulomi S, Borges AV, Abril G, Bouillon S (2008). Emission of CO2 and CH4 to the atmosphere by sediments and open waters in two Tanzanian mangrove forests. *Mar Ecol Prog Ser* **370:** 53-67.

Krithika K, Purvaja R, Ramesh R (2008). Fluxes of methane and nitrous oxide from an Indian mangrove. *Current Science* **94:** 218-224.

Livesley SJ, Andrusiak SM (2012). Temperate mangrove and salt marsh sediments are a small methane and nitrous oxide source but important carbon store. *Estuarine, Coastal and Shelf Science* **97:** 19-27.

Marinho C, Campos E, Guimarães J, Esteves F (2012). Effect of sediment composition on methane concentration and production in the transition zone of a mangrove (Sepetiba Bay, Rio de Janeiro, Brazil). *Brazilian Journal of Biology* **72:** 429-436.

Queiroz HM, Artur AG, Taniguchi CAK, Silveira MRSd, Nascimento JCd, Nóbrega GN *et al* (2019). Hidden contribution of shrimp farming effluents to greenhouse gas emissions from mangrove soils. *Estuarine, Coastal and Shelf Science* **221:** 8-14.

Rosentreter JA, Maher DT, Erler DV, Murray R, Eyre BD (2018). Factors controlling seasonal CO2 and CH4 emissions in three tropical mangrove-dominated estuaries in Australia. *Estuarine, Coastal and Shelf Science* **215:** 69-82.

Santos IR, Maher DT, Larkin R, Webb JR, Sanders CJ (2019). Carbon outwelling and outgassing vs. burial in an estuarine tidal creek surrounded by mangrove and saltmarsh wetlands. *Limnol Oceanogr* **64:** 996-1013.

Sea MA, Garcias-Bonet N, Saderne V, Duarte CM (2018). Carbon dioxide and methane fluxes at the air–sea interface of Red Sea mangroves. *Biogeosciences* **15:** 5365-5375.

Zheng X, Guo J, Song W, Feng J, Lin G (2018). Methane Emission from Mangrove Wetland Soils Is Marginal but Can Be Stimulated Significantly by Anthropogenic Activities. *Forests* **9:** 738.
