# Supplementary figures and images for: Genomic and transcriptomic insights into methanogenesis potential of novel methanogens from mangrove sediments
Source: Microbiome. 2020 Jun 17;8:94. doi: 10.1186/s40168-020-00876-z (PMC7302380; doi:10.1186/s40168-020-00876-z)

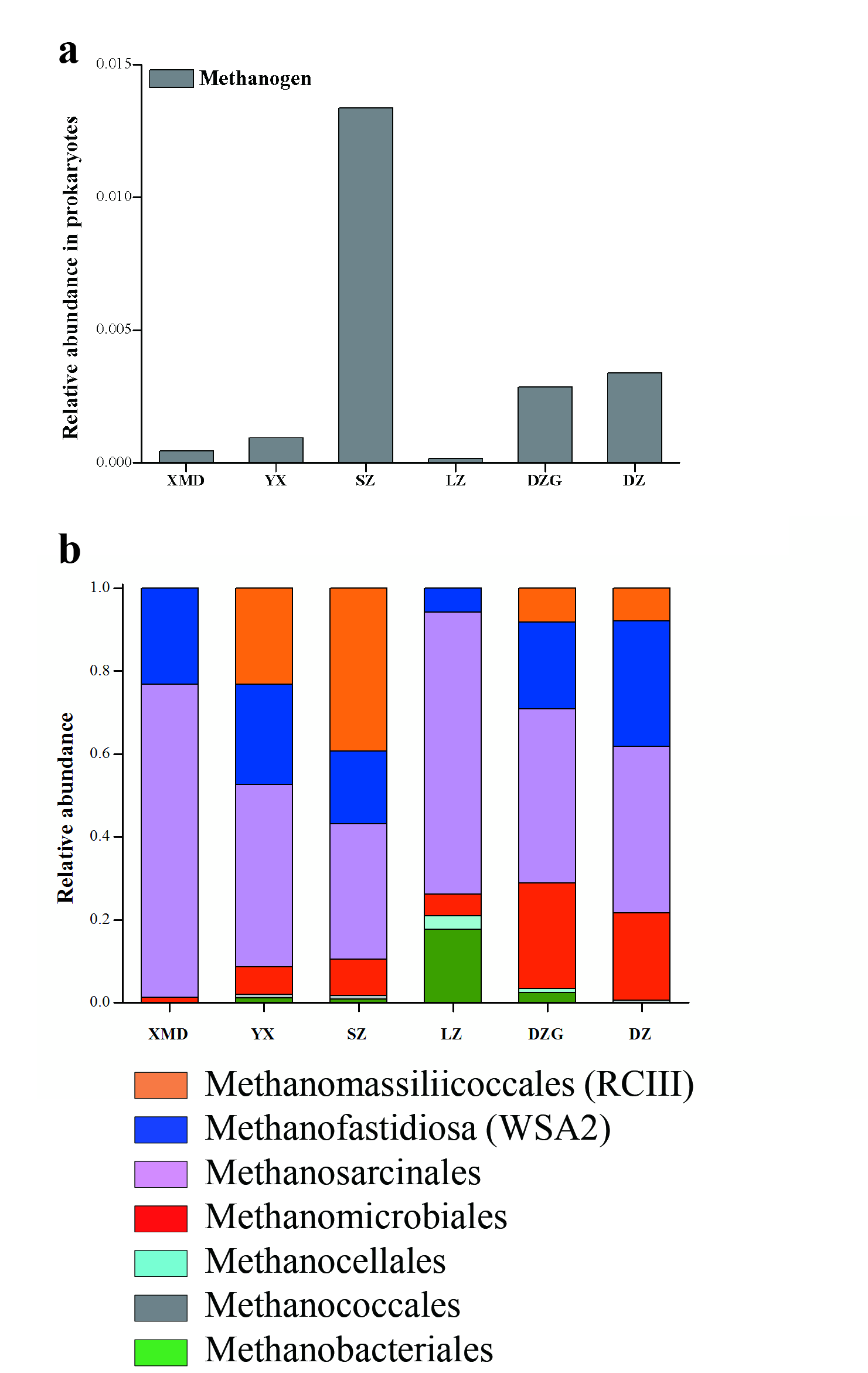

Supplement: Supplementary file 3 — Additional file 2: Figure S1. Relative abundance of methanogens in prokaryotes (a) and community composition of methanogens (b) among 6 mangroves across southeastern China. [file 40168_2020_876_MOESM2_ESM.tif]

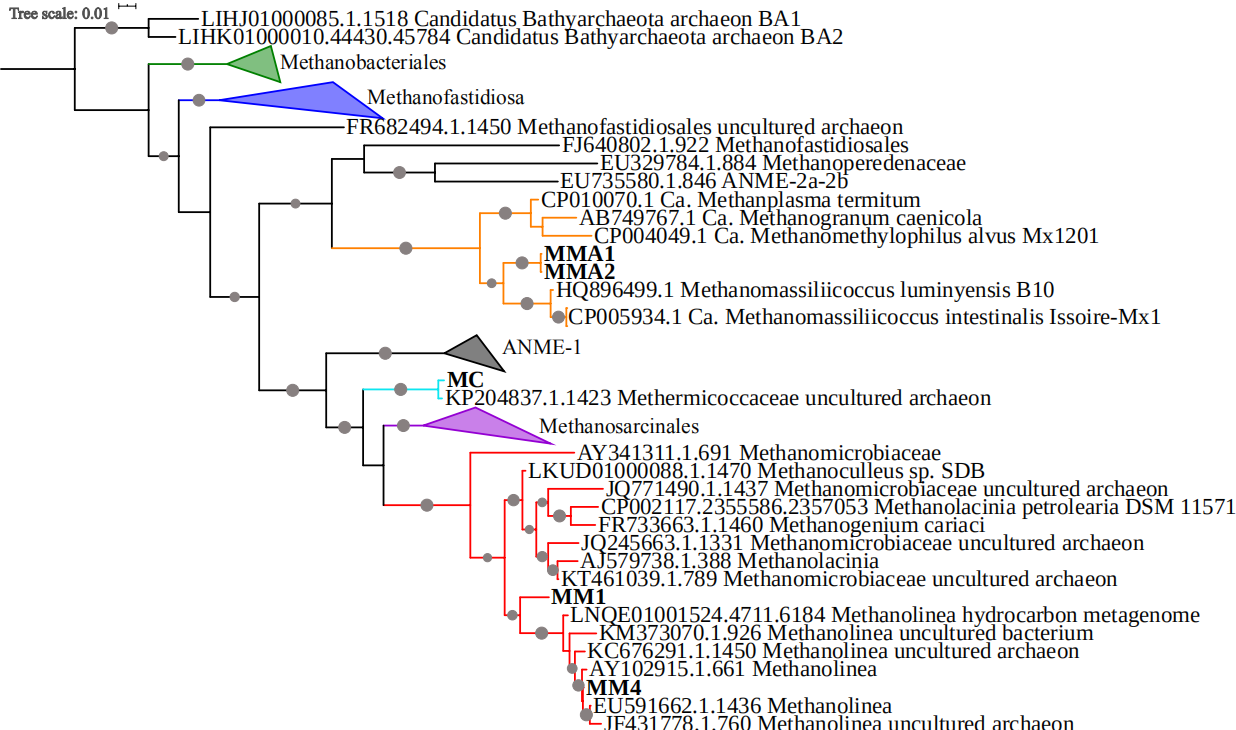

Supplement: Supplementary file 4 — Additional file 3: Figure S2. Phylogenetic trees of recovered MAGs using 16S rRNA gene sequences. Each name in bold represent a MAG. Bootstrap values were calculated via non-parametric bootstrapping with 100 replicates, and are represented by grey circles in different sizes. The scale bar indicates 10% estimated phylogenetic divergence. [file 40168_2020_876_MOESM3_ESM.tif]

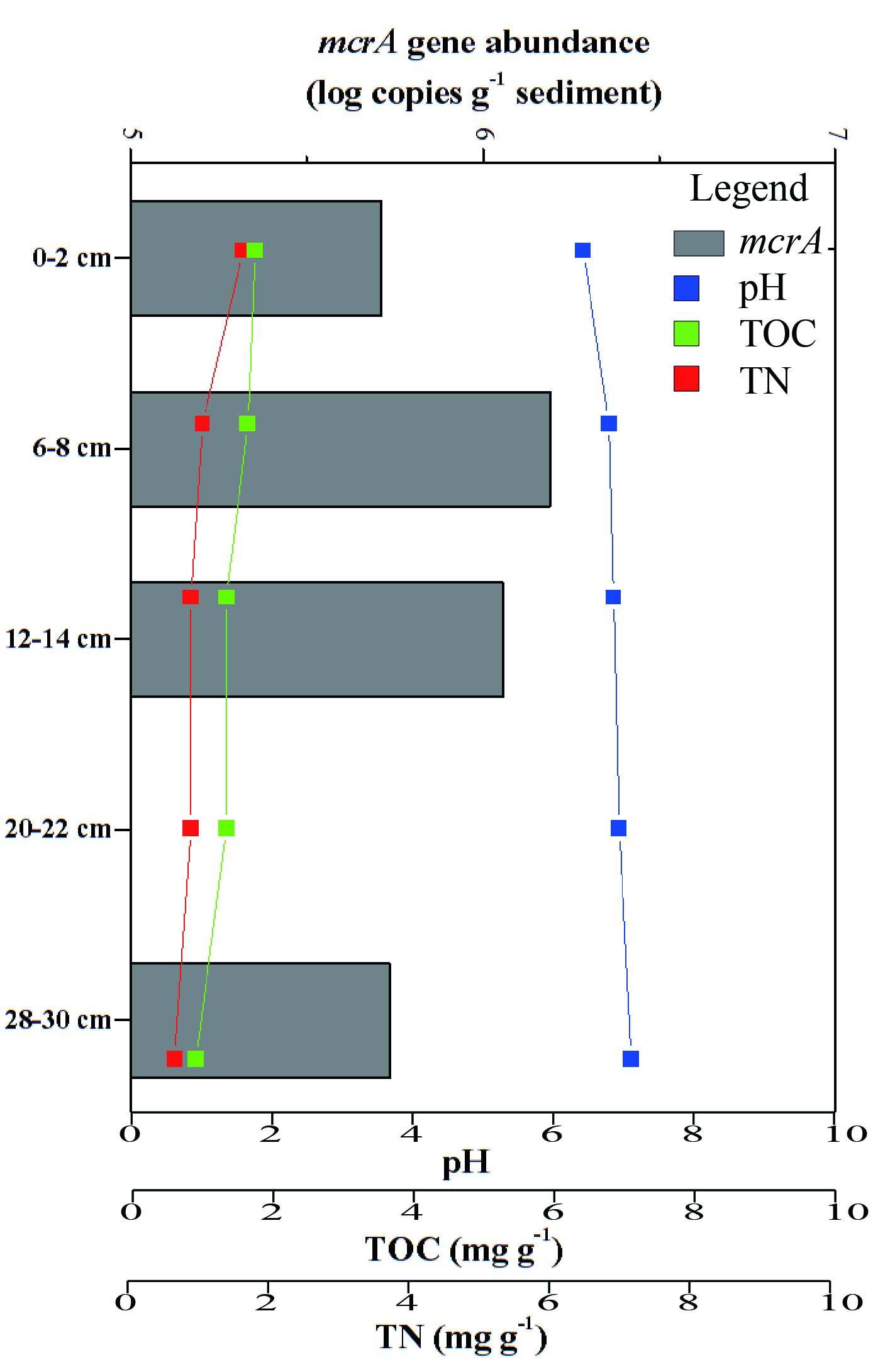

Supplement: Supplementary file 5 — Additional file 4: Figure S3. Physiochemical properties and mcrA gene copies in five layers of mangrove sediments. [file 40168_2020_876_MOESM4_ESM.tif]

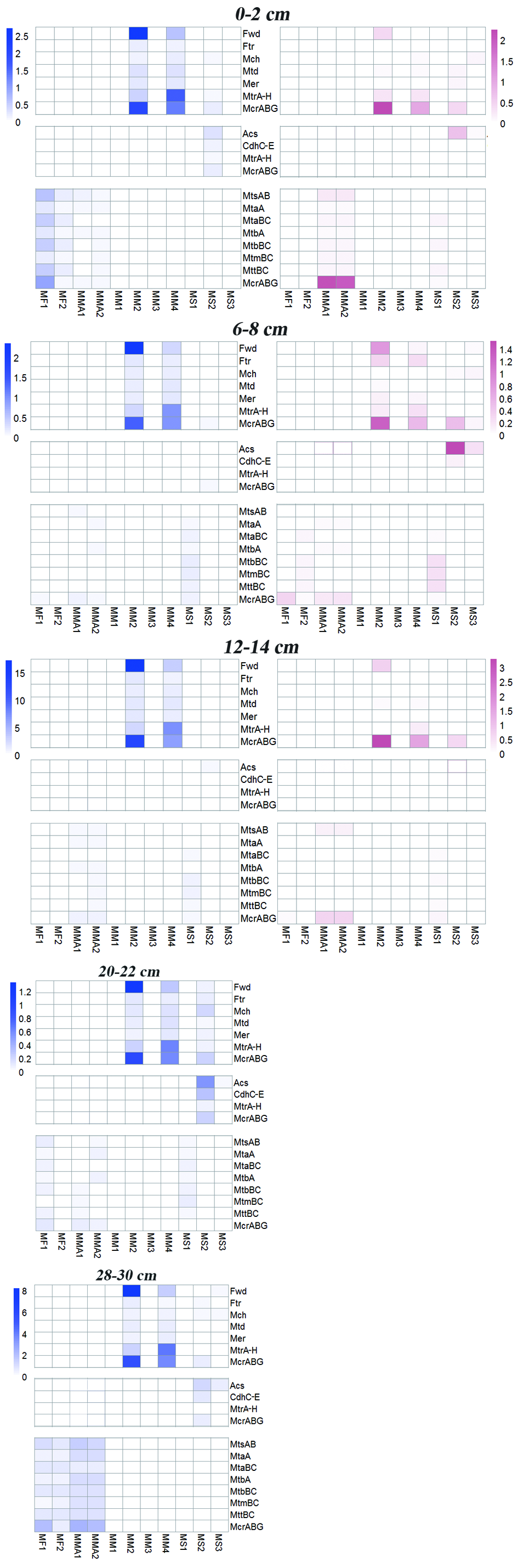

Supplement: Supplementary file 6 — Additional file 5: Figure S4. The relative abundances (metagenome, FPKM, blue) and expression levels (metatranscriptome, FPKM, red) of genes involved for methanogenesis with affiliation to four dominant methanogens (MF, MMA, MM and MS) at five layers in mangrove sediments. [file 40168_2020_876_MOESM5_ESM.tif]
